# Performance evaluation of a laboratory developed PCR test for quantitation of HIV-2 viral RNA

**DOI:** 10.1371/journal.pone.0229424

**Published:** 2020-02-28

**Authors:** Linda L. Jagodzinski, Mark M. Manak, Holly R. Hack, Ying Liu, Sheila A. Peel

**Affiliations:** 1 U.S. Military HIV Research Program, Walter Reed Army Institute of Research, Silver Spring, Maryland, United States of America; 2 U.S. Military HIV Research Program, Henry M. Jackson Foundation for the Advancement of Military Medicine, Bethesda, Maryland, United States of America; CEA, FRANCE

## Abstract

Management of Human Immunodeficiency Virus Type 2 (HIV-2) infections present unique challenges due to low viral titers, slow disease progression, and poor response to standard antiviral therapies. The need for a nucleic acid assay to detect and quantify HIV-2 virus has led to the development of a number of molecular-based assays for detection and/or quantification of HIV-2 viral RNA in plasma in order to provide laboratory evidence of HIV-2 infection and viral loads for use in treatment decisions. As HIV-2 is less pathogenic and transmissible than HIV-1 and has resistance to several of the antiretroviral drugs, delay of treatment is common. Cross sero-reactivity between HIV-1 and HIV-2 makes it difficult to distinguish between the two viruses based upon serological tests. As such we developed a quantitative reverse transcription PCR (qRT-PCR) assay targeting the 5’ long terminal repeat of HIV-2 for detection and quantification of HIV-2 viral RNA in plasma to identify HIV-2 infection and for use in viral load monitoring. Serial dilutions of cultured HIV-2 virus demonstrated a wide dynamic range (10 to 100,000 copies/ml) with excellent reproducibility (standard deviation from 0.12–0.19), linearity (*R*^2^ = 0.9994), and a lower limit of detection at 79 copies/ml (NIH-Z). The assay is highly specific for HIV-2 Groups A and B and exhibits no cross reactivity to HIV-1, HBV or HCV. Precision of the assay was demonstrated for the High (Mean = 6.41; SD = 0.12) and Medium (Mean = 4.46; SD = 0.13) HIV-2 positive controls. Replicate testing of clinical specimens showed good reproducibility above 1,000 copies/ml, with higher variability under 1,000 copies/ml. Analysis of 220 plasma samples from HIV-2 infected West African individuals demonstrated significantly lower viral loads than those observed in HIV-1 infections, consistent with results of previous studies. Slightly more than seven percent of clinical samples (7.3%) demonstrated viral loads above 100,000 copies/ml, while 37.3% of samples were undetectable. The high sensitivity, specificity, precision, and linearity of the WRAIR qRT-PCR assay makes it well suited for detection and monitoring of HIV-2 RNA levels in plasma of infected individuals.

## Introduction

Human immunodeficiency virus (HIV) is categorized into two types, HIV-1 and HIV-2, with approximately 1–2 million of the 37 million HIV infections worldwide attributed to HIV-2; Groups A and B are the most common [1–3]. While HIV-2 is largely confined to regions of West Africa, the infection has also been identified in India, Western Europe, Brazil, the Caribbean and other regions [4]. HIV-2 infections are relatively rare in the US, with highest infection rates in the Northeast (including in New York City) and among persons born in West Africa [5, 6]. HIV-2 infected persons are often aviremic, or have lower viral loads (VL) thus lower transmission potential, and a slower disease progression than those infected with HIV-1, with only about 20 to 30% of infected persons ultimately progressing to acquired immunodeficiency syndrome AIDS [7–11]. Left untreated, however, infection with HIV-2 can lead to continued risk of HIV transmission, higher cost of medical management, and increased mortality [12, 13]. Standard HIV antiretroviral therapies (ART) are often ineffective for treatment of HIV-2 due to drug resistance that results in high rates of virologic failures despite good treatment adherence [14–20]. The limited HIV-2 therapeutic arsenal, frequent cross-resistance, and incomplete knowledge about resistance pathways dramatically limits treatment options [21–23].

The current HIV Diagnostic Algorithm recommended by the CDC requires screening of samples by the HIV-1/-2 Ag/Ab combo assay and differentiation of HIV-1 and HIV-2 infections by a type specific Assay (Geenius HIV-1/2 Supplemental Assay, BioRad) with discrepant samples resolved by RNA testing. However, since there are no CE Marked nor U.S. FDA cleared assays for HIV-2 viral RNA, many laboratories have developed their own HIV-2 RNA assay or send discrepant samples to a national reference laboratory for resolution [24–29]. Furthermore, the WHO consolidated guidelines on the use of antiretroviral drugs for treating and preventing HIV infection recommend use of plasma viral load for monitoring of antiretroviral therapy and disease progression [30]. Due to low viremia and slow disease progression people living with HIV-2 tend to initiate ART later than those with HIV-1, and providers are faced with unique challenges in management of their infections. Several laboratories have developed quantitative HIV-2 viral RNA assays to provide information on the levels of virus in the plasma [24–26, 28, 31, 32]. The Long Terminal Repeat (LTR) or the *gag* gene regions are commonly targeted as they are more conserved than other regions of the virus. The University of Washington (Seattle, WA) uses an in-house HIV-2 qRT-PCR assay targeting the LTR that has been validated according to College of American Pathology (CAP) regulations on the Abbott m2000sp/rt platform [24]. The Wadsworth Center (New York State Department of Health) performs HIV-2 RT digital droplet PCR (ddPCR) testing under CAP [25]. The Public Health Agency of Canada (Winnipeg, MB) also offers a HIV-2 RT-ddPCR assay. Several commercial companies, including Primer Design LTD, Southampton, UK (Genesig Advanced Real-Time PCR HIV-2 Detection kit)) and Biocentric, Bandol, France (GENERIC HIV-2 qRT-PCR) have Research Use Only (RUO) HIV-2 RNA kits or assays available in Europe. This paper describes the Walter Reed Army Institute of Research (WRAIR) quantitative reverse transcription real-time PCR (WRAIR qRT-PCR) assay used for testing of specimens received from Military Testing Facilities [33]. The assay was evaluated for linearity, sensitivity, specificity, limit of detection (LOD), and precision for use in clinical confirmation of HIV-2 infection status and for monitoring of HIV-2 plasma viral loads of infected individuals in the U.S. Military and their Beneficiaries.

## Materials and methods

### HIV-2 calibration standard

A recombinant plasmid clone containing a 795 bp insert of HIV-2 LTR/*gag* gene regions (LTR556/GAG1319) was constructed in the TA cloning vector pCR4-TOPO. The plasmid was linearized downstream from the insert using Not I restriction enzyme. The linearized HIV-2 LTR/*gag* plasmid DNA was used as a template for RNA transcription using Ambion’s MEGAscript T7 Transcription kit (AM1333: Thermo Fisher Scientific, Life Technologies, Carlsbad, CA) following procedures recommended by the manufacturer. The quality of the HIV-2 RNA transcript (865 bases) was confirmed by electrophoretic fractionation through a 2% RNA agarose gel. RNA transcripts were purified using MEGAclear Transcription Clean-up kit (AM1908: Thermo Fisher Scientific, Inc., Carlsbad CA) and quantified by optical density absorbance readings using the Nanodrop 2000 (Thermo Fisher Scientific, Inc., Waltham, MA). A mean value from three replicate concentrations was converted to copies/ml using Avogadro’s number.

A working stock of the HIV-2 RNA transcript was prepared at 1x10^8^ HIV-2 RNA copies/ml in 1 μg/ml tRNA diluent (AM7119: Ambion, Thermo Fisher), aliquoted at 25–100 μl per tube and stored at <-70°C. Calibration standards were prepared by 10-fold serial dilutions of HIV-2 RNA working stock in tRNA diluent. Ten microliters of each calibration standard starting with the working stock were added to 15 μls of the RT-PCR reaction mix to obtain HIV-2 RNA input copies at 1x10^1^, 1x10^2^, 1x10^3^, 1x10^4^, 1x10^5^ and 1x10^6^ and were included in each test run. The 10 copy calibrator was tested in triplicate as amplification at this value varied and was not always detected in the assay. The cycle threshold (C_*T*_) values of the calibrators were used to assign HIV-2 RNA copies. Samples which failed to generate a C_*T*_ value were designated as target not detected (TND).

### Internal extraction control

A well-characterized stock of Bacteriophage MS2 (ATCC, Manassas, VA) was used as an Internal Extraction Control (IC) to monitor the efficiency of extraction, reverse transcription, and amplification reactions. The concentration of the MS2 phage was adjusted to provide a C_*T*_ value of 24–27, a level selected to limit competition with the HIV-2 target. The MS2 Internal Control (IC) was added to the lysis buffer and co-purified with the HIV-2 RNA for each sample. Amplification of MS2 IC was performed in a separate reaction well at the same time and conditions as the HIV-2 qRT-PCR [33, 34].

### HIV-2 controls, and panels for limit of detection (LOD) and analytical measurement range (AMR)

HIV-2 RNA positive controls were prepared from a culture stock of HIV-2 NIH-Z (Group A: Advanced BioTechnologies, Columbia, MD) whose concentration was determined by Electron Microscopy particle count. Serial dilutions of the HIV-2 NIH-Z stock were used to establish assay linearity and prepare medium and high extraction controls targeted at 5,000 to 20,000 and 500,000 to 2,000,000 copies/ml, respectively. A three (3) to four (4) C_*T*_ acceptability range was established with the medium control having a C_*T*_ value from 25 to 29 and the high from 19 to 22. The negative plasma control was either BaseMatrix, a defibrinated, dialyzed plasma product (SeraCare, Milford, MA) or pathogen free normal human plasma (NHP) which had been prescreened as negative for HIV, HBV and HCV serological and nucleic acid markers. LOD and AMR panels were prepared by serial dilutions of the NIH-Z viral stock into BaseMatrix to generate HIV-2 viral concentrations at 500,000, 50,000, 5,000, 1,000, 500, 250, 100, 50 and 25 copies/ml. Controls and panel members used in this study were aliquoted at 0.5 ml for single use and stored at <-70°C.

### Viral stocks and World Health Organization (WHO) HIV-2 RNA International Standard (IS)

Ten HIV-2 viral stocks (9 Group A and 1 Group B) were obtained from the NIH AIDS Reagent Program (www.aidsreagent.org). An additional HIV-2 Group B viral culture stock was purchased from SeraCare, Inc. (Medford, MA). HIV-1 subtypes A, B, C, D, CRF01_AE and CRF02_AG were selected from the WRAIR HIV-1 Subtype Panel [35, 36]. HIV-1, HCV and HBV high viral controls were purchased from Acrometrix (Benicia, CA). The WHO HIV-2 RNA IS (08/150) was obtained from the National Institute for Biological Standards and Control (NIBSC: South Mimms, Potters Bar, UK) [37]. The WHO HIV-2 IS was provided as freeze-dried material and was reconstituted per instructions to provide an HIV-2 standard at 1,000 International Units (IU)/ml [37]. A higher titer HIV-2 Working Reagent material was also provided by NIBSC for HIV-2 RNA assessment (Clare Morris, NIBSC).

### Clinical samples

An HIV-2 Performance Panel (PRF203) consisting of one HIV-2 negative plasma sample (panel member 11) and ten clinical specimens reactive by the Abbott HIV Ag/Ab combo, with specific reactivity for HIV-2 by HIV-1/HIV-2 differentiation assays was purchased from SeraCare, Inc. HIV-2 viral RNA values were provided by SeraCare based upon a laboratory developed qRT-PCR assay targeting the HIV-2 *gag* gene region with a synthetic RNA calibrator used for quantitation [38]. Twelve HIV-2 serologically positive clinical samples were also purchased from Boca Biolistics (Pompano Beach, FL). EDTA whole blood from HIV negative donors (N = 50) and pathogen free EDTA plasma (N = 21) were obtained from Biological Specialty Laboratories, Inc. (Comar, PA). Two hundred and twenty (220) clinical plasma specimens from Cote d’Ivoire which were reactive in the HIV1/2 Ag/Ab Combo assay and confirmed as HIV-2 reactive by either the Bio-Rad Genie II EIA HIV-1/HIV-2 or Determine HIV-1/HIV-2 Ab/Ab Combo assays or by HIV-2 RNA assay were provided under a Collaborative Research and Development Agreement with SeraCare, Inc. (Milford, MA). All samples were serologically non-reactive for HIV-1 in the HIV-1/HIV-2 differentiation assays. Eight clinical samples were non-reactive in the HIV-2 differentiation assay; but, had quantified HIV-2 viral RNA values ranging from 20 to 50,000 HIV-2 RNA copies/ml as measured by independent quantitative HIV-2 RNA assays as reported by SeraCare, Inc. These eight samples are believed to be from individuals in early infection based upon non-reactivity in the HIV-1/HIV-2 differentiation assays and a measured viral load value. The HIV-2 viral RNA assay was noted as University of Washington for six of the eight samples and H**ô**pital Bichat-Claude Bernard in Paris for two samples.

### RNA extraction

RNA was purified from 0.4 ml of plasma using the QIAamp MinElute Virus Spin kit (Qiagen, Valencia, CA) and eluted in 50 μl of AVE buffer according to the manufacturer’s recommendations. The MS2 phage, internal extraction control, was added to the sample lysis buffer prior to extraction. The purified RNA was used within 30 minutes of completed extraction or stored at <-70°C.

### Primers and probes

HIV-2 sequence-specific primers and probe were designed to amplify 134 base pairs of the conserved 5’ long terminal repeat region (LTR, R/U5) using Allele ID 7.0 Species Specific TaqMan®/ MGB Assay (PREMIER Biosoft International), Primer Express (Applied Biosystems, Foster City, CA), Primer Design (Educational software, NC), and Integrated DNA Technologies (IDT, Coralville, IA) software packages. Primers and probe sequences were selected from regions having the least number of mismatched bases for HIV-2 Group A and B sequences (HIV Sequence Database, Los Alamos National Laboratory, Los Alamos, NM). Amplification across the R/U5 junction allowed for amplification of a single target per HIV-2 viral RNA. In order to decrease the potential for base pair mismatches the length of the primers and probes were kept under 20 bases. Inosine was used in the forward primer to allow for detection of HIV-2 variants. The HIV-2 probe was designed as a Major Grove Binding (MGB) probe in order to increase the probe melting temperature and improve binding.

The HIV-2 nucleotide sequences and locations are provided with respect to SMM239 (M33262: https://www.hiv.lani.gov/content/sequence/HIV): forward primer, CCTGCTIGACTCTCACC (596→612); reverse primer, GCGGCGACTAGGAGAGAT (712←729); and probe, 6-FAM-CCACGCTTGCTTGCTT-MGBNFQ (643→658). HIV-2 LTR and MS2 phage primers and MS2 probe were synthesized by IDT (Coralville, IA). The MS2 probe was labelled with JOE at the 5’-end and quenched with BHQ1 at the 3’-end to allow for simultaneous amplification of the HIV-2 and MS2 targets. The HIV-2 MGB probe was synthesized by Applied Biosystems (Foster City, CA). The concentration of the primers and probe stocks were determined by absorbance at 260/280 nm using Nanodrop 2000 and diluted to 100 μM for primers and 50 μM for probes.

### Real-time RT-PCR amplification

The Superscript III Platinum One-Step Quantitative RT-PCR kit (Thermo Fisher Scientific, Life Technologies, Carlsbad, CA) was used for amplification reactions. The 25 μl reaction volume consisted of 1X reaction buffer, 0.6 μM forward and reverse primers, 0.2 μM probe, Reverse Transcriptase (RT) and DNA polymerase enzymes and 10 μl of RNA. Real-time amplification was performed on the 7500 Fast Dx instrument (Thermo Fisher Scientific, Life Technologies) at the following cycling parameters: 1) reverse transcription at 50°C for 30 minutes, 2) activation of the DNA polymerase at 95°C for 2 minutes, 3) 5 cycles of amplification at 95°C for 15 seconds, 52°C for 10 seconds, and 60°C for 1 minute, and 4) 40 cycles of amplification at 95°C for 15 seconds, 57°C for 10 seconds, and 60°C for 1 minute with fluorescent read. The initial 5 cycles of amplification under less stringent annealing conditions followed by 40 cycles at increased stringency (Step-up method) allowed for improved sensitivity across regions containing minor mismatches.

Assay acceptance criteria required the calibration curve to have a slope from -3.0 to -3.6 with a *R*^2^ >0.95; not detected HIV-2 results for the negative and no template controls (NTC); MS2 C_*T*_ values between 24 to 27 for all RNA extractions; and HIV-2 C_*T*_ values between 19 to 22 for the high control and 25 to 29 for the medium control. Runs which failed to achieve these criteria were rejected and the test repeated. The assay uses 400 μl of plasma sample with elution of the purified RNA in 50 μl of which 10 ul is used in the amplification (equivalent of 80 μl of sample input per assay). The HIV-2 RNA assay copy number was extrapolated from the HIV-2 calibration standard curve by the software program and multiplied by 12.5 (1000 μl/80 μl) to convert HIV-2 copies/assay to copies/ml.

The Genesig Advanced Real-Time PCR HIV-2 Detection kit (Primer Design LTD, Southampton, UK) assay was performed as previously described (31), except for the use of the NIH-Z viral stock instead of the HIV-2 DNA calibrator included in the kit. Amplification cycling parameters for this assay were 50°C for 20 minutes (reverse transcription), 95°C for 2 minutes (enzyme activation) and 45 cycles at 95°C for 10 second, 57°C for 10 seconds and 60°C for 1 minute (fluorescent read).

### Statistical analysis

Data analysis was performed using Microsoft Excel and Prism 7 (GraphPad Software, La Jolla, CA) for calculation of correlation coefficient, linear regression, means, ratios, and standard deviation. Coefficient of Variance (%CV) was also calculated according to the formula provided in Canchola, etal. for log-transformed data [39]. The Limit of Detection was determined by graphical analysis in Prism comparable to Probit.

### Regulatory approval

Clinical specimens used in this study were received from commercial sources with no personal identifiers. The Walter Reed Army Institute of Research (WRAIR) Human Subjects Protection Branch determined this study (WRAIR #1404 –RV234, sub-study #7) to be non-human subjects research (NHSR) not requiring Institutional Review Board approval based on the analysis of anonymized samples with no link to specific individuals.

## Results

### Assay calibration

**Fig 1:**
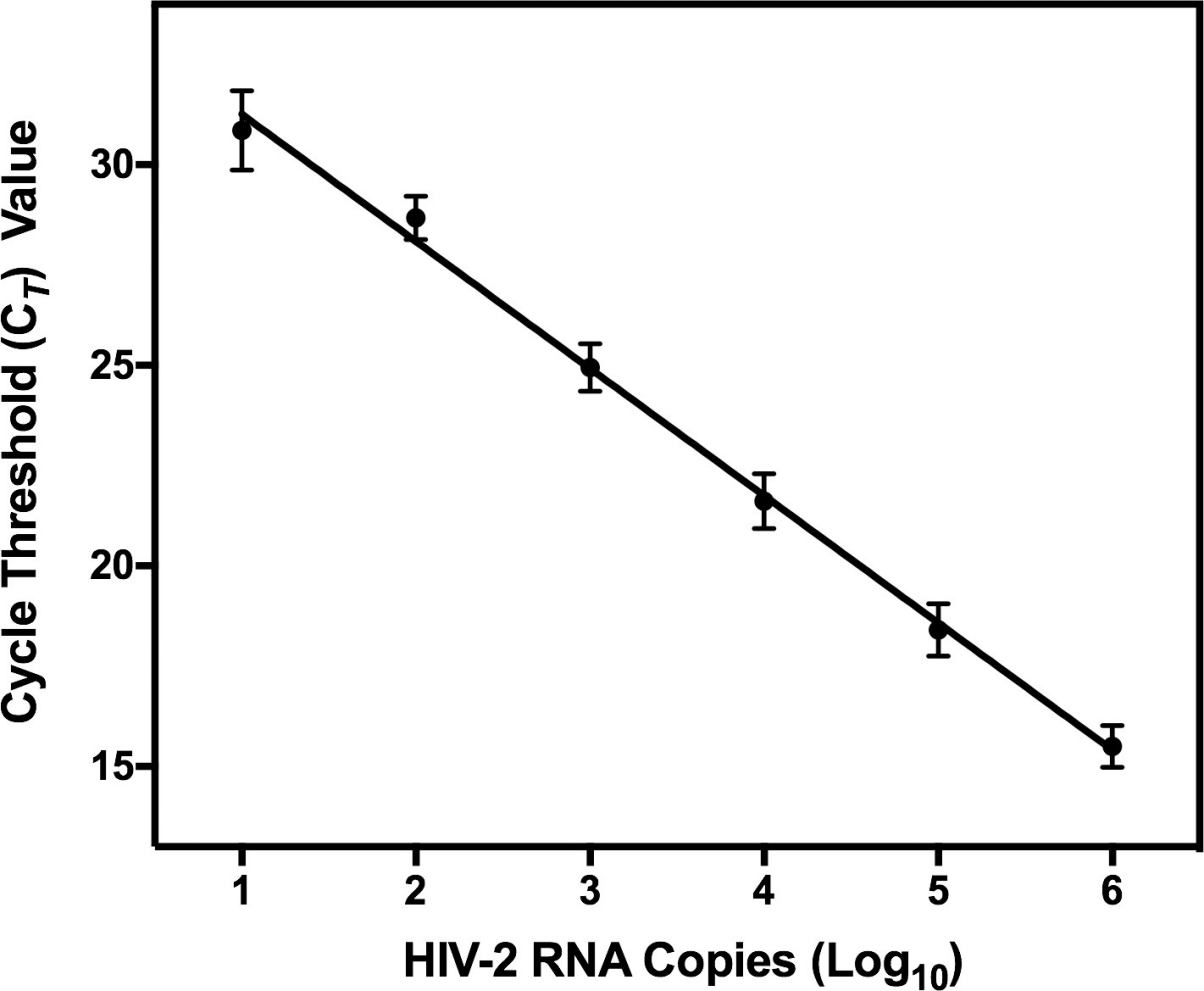
HIV-2 RNA calibration curve. The HIV-2 RNA standard was 10-fold serially diluted and amplified in each assay run. HIV-2 RNA copy number was extrapolated from the calibration curve generated for the HIV-2 RNA calibrators based on the C_*T*_ values. Average C_*T*_ values from 44 runs of the assay showed excellent linearity (*R*^2^ = 0.997) and precision (1.88–3.47% CV) between 2.0 and 6.0 log_10_ HIV-2 RNA input copies.

Each run of the WRAIR qRT-PCR assay included a series of calibrators ranging from 10 to 1,000,000 HIV-2 RNA input copies. The assay showed a broad linear dynamic range from 10 to 1,000,000 input copies with an *R*^2^ = 0.997 (Fig 1). The calibrator C_*T*_ values showed excellent precision over 44 runs ranging from 1.88% to 3.47% CV. The 10 copy calibrator showed more variability and was tested in triplicate in each run, with 40 of 70 replicates detected.

### RNA recovery

**Fig 2:**
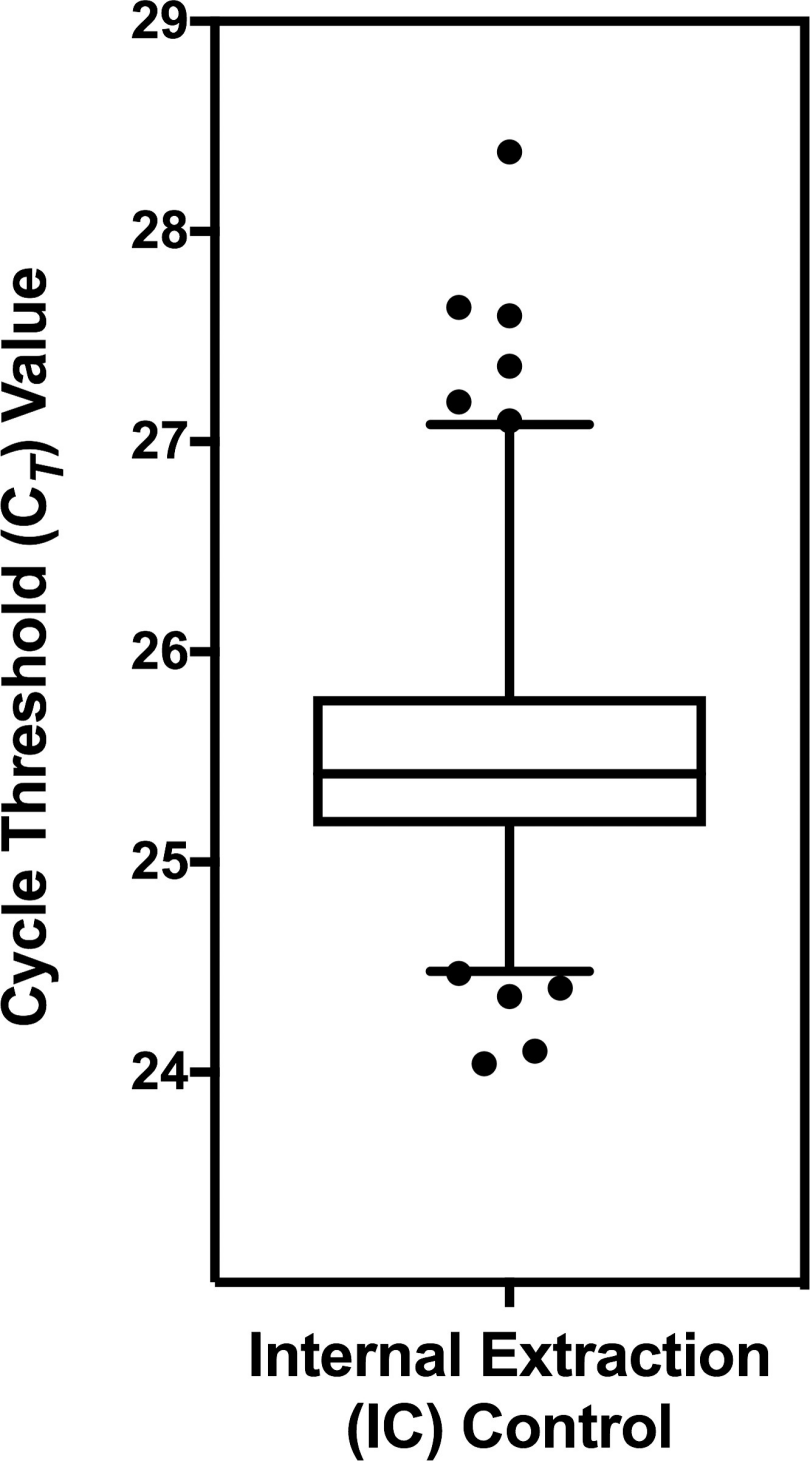
Performance of MS2 phage internal control (IC). Box and Whisker plot for a total of 647 replicates of the IC over multiple runs showing the mean (25.35 +/- 0.41) and 25/75 percentiles (box).

An internal control, consisting of MS2 phage (RNA) was added to each sample during extraction to monitor recovery and amplification efficiency. Fig 2 shows a Box and Whisker plot of the mean (25.35) and 25–75 percentile of C_*T*_ values of the Internal Control in 647 reactions. Samples in which the C_*T*_ values of the IC were more than 3 SD above the mean (>27 C_*T*_) were considered indicative of poor sample recovery or the presence of inhibitors which led to decreased amplification efficiency, and were designated as invalid, requiring repeat testing.

### Assay precision

**Fig 3:**
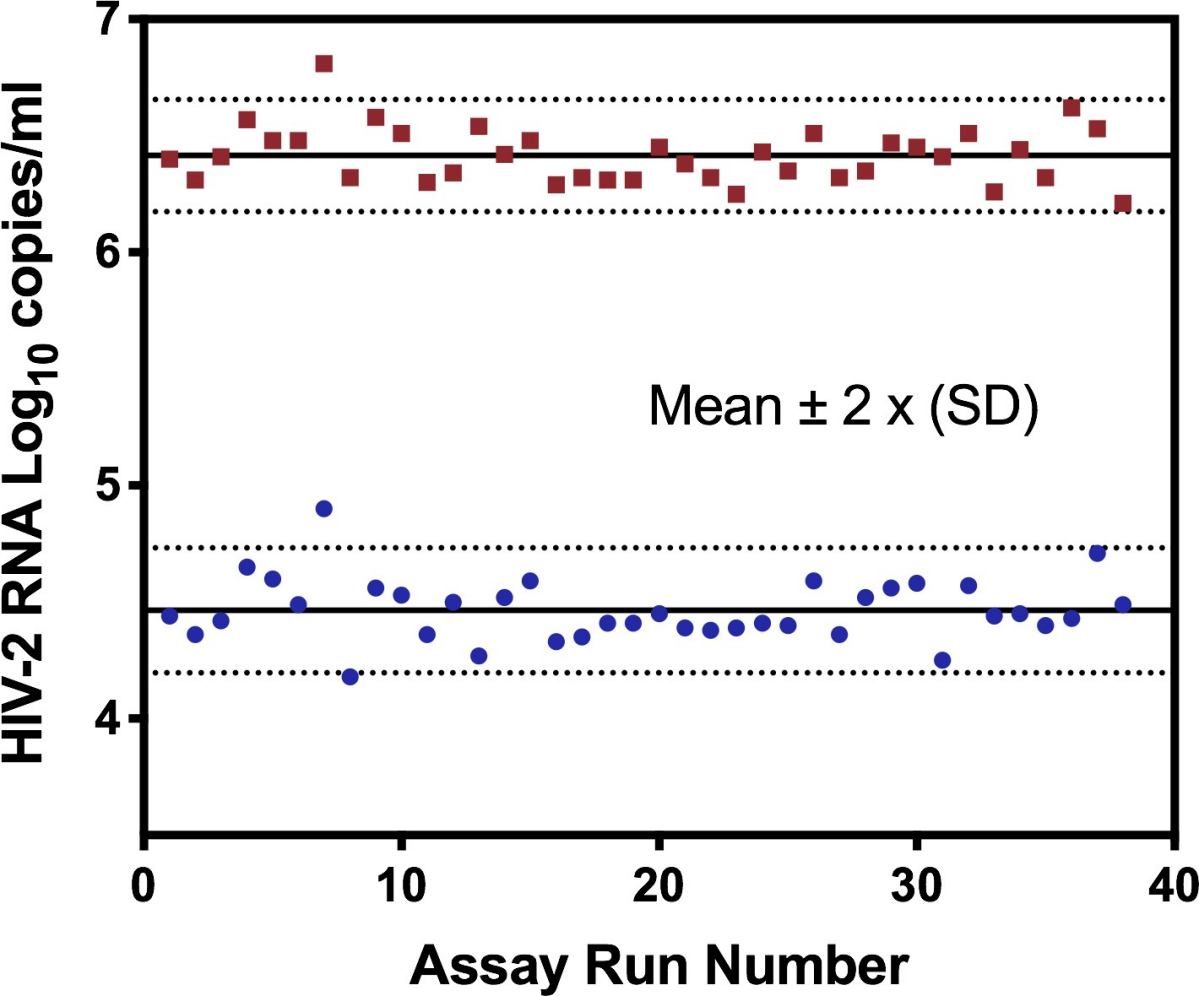
Precision of HIV-2 positive extraction run controls. The precision of the high and medium extraction controls is shown in a Levey-Jennings plot for 38 assay runs on log_10_-transformed values. Solid lines indicate means with dotted lines at +/- 2 standard deviations (SD).

The precision of the WRAIR qRT-PCR assay was evaluated based on results of the Medium and High Extraction controls included in each test run. A Levey-Jennings analysis of 38 separate runs showed that the HIV-2 values for the High and Medium Run Controls were consistent between runs with an average of 6.41 +/- 0.12 and 4.46 +/- 0.13 log_10_ copies/ml (Fig 3). Coefficient of variance for non-transformed values were 32.23% and 36.43% for High and Medium Run controls. No amplification signal was detected for any of the negative run controls (N = 38) and No Template Controls (NTC) whose results were reported as “Target Not Detected” (TND).

### Linearity

**Fig 4:**
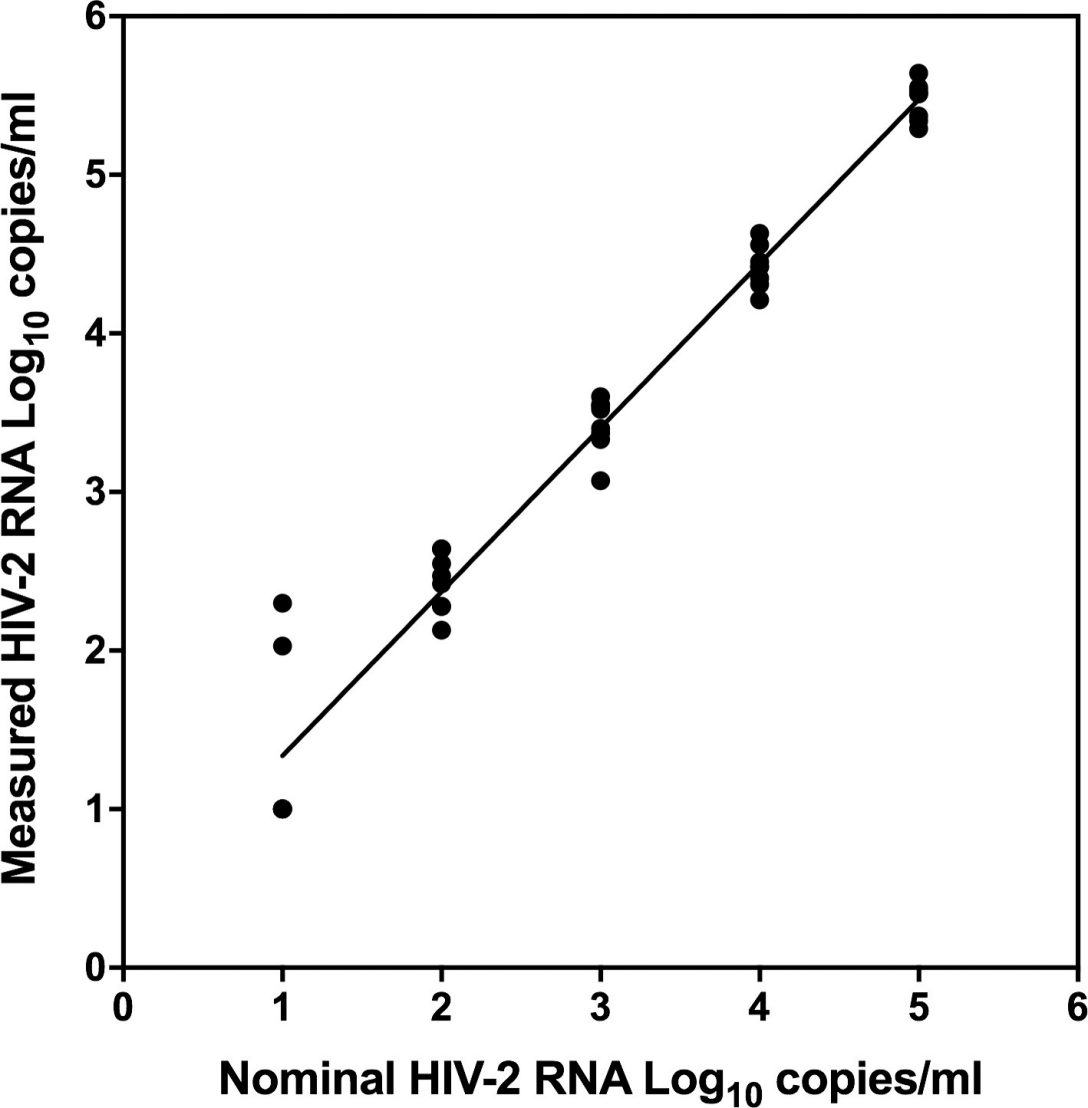
Linearity of the WRAIR qRT-PCR assay. Ten-fold serial dilutions of the HIV-2 CAM-2 isolate in normal human plasma ranging from 1.0 to 5.0 HIV-2 RNA log_10_ copies/ml were tested in replicates in separated assay runs to provide results for 8 replicates at each concentration. HIV-2 viral RNA values generated demonstrated excellent linearity across the range of concentrations tested. *R*^2^ = 0.9994. Y = 1.035*X + 0.3018.

The linearity of the WRAIR qRT-PCR assay was evaluated on serial dilutions of the HIV-2 Working Reagent (CAM-2 isolate) kindly provided by NIBSC. Two separate sets of 10-fold serial dilutions of the virus in NHP were tested in duplicate at 100,000 to 10 copies/ml over two days for a total of 8 replicates at each dilution. The results show excellent linearity with an *R*^2^ of 0.9994 and high reproducibility (SD 0.12–0.19) between 2.0 and 5.0 log_10_ copies/ml (Fig 4). Three of eight replicates were detected at the 10 copies/ml dilution.

### Subtype specificity and sensitivity

HIV-2 subtype specificity was evaluated with a series of HIV-1 and HIV-2 cultured isolates, and high viral load HBV and HCV human plasma samples. The assay yielded results in the expected range when testing HIV-2 isolates, including nine Group A and two Group B isolates, and did not detect any of the HIV-1 subtypes tested including subtypes A, B, C, D, CRF01_AE, and CRF02_AG (Table 1). The assay was also negative on commercially acquired plasma controls containing high viral loads of HIV-1, HBV, and HCV. These results indicate that the WRAIR qRT-PCR assay is specific for HIV-2 and is not cross-reactive with HIV-1, HBV or HCV. Plasma samples from uninfected individuals (N = 21) and for three lots of negative plasma controls were consistently not detected.

**Table 1:** WRAIR qRT-PCR assay specificity and sensitivity.

			Log_10_(Copies/mL)	
	Analyte or Isolate	Group Subtype	Target Value	Result
	CDC 77618	A	~ 6.5	6.24
	CBL-23	A	~ 6.5	7.13
	CBL-20	A	~ 6.5	6.62
	MVP-15132	A	~ 6.5	6.97
	D194	A	~ 6.5	6.44
**HIV-2**	7924A	A	~ 6.5	6.14
	60415K	A	~ 6.5	6.17
	CDC 310248	A	~ 6.5	5.94
	CDC 310072	A	~ 6.5	6.53
	PB0012902206	B	~ 6.5	5.50
	CDC 310319	B	~ 6.5	6.20
	KSM4030E5	A	5.00	TND
	90US873E5	B	5.00	TND
**HIV-1**	02ET14E6	C	5.00	TND
	E08364M4E5	D	5.00	TND
	NO1251E5	CRF01_AE	5.00	TND
	BC132E5	CRF02_AG	5.00	TND
	HIV-1	B	5.70	TND
**Acrometrix**	HBV	-	6.70	TND
	HCV	-	5.70	TND
**Neg**	NHP	-	-	TND

NHP = Pathogen free Normal Human Plasma

TND = Target Not Detected

### Assay standardization

The WHO HIV-2 International Standard (IS) was used to establish a conversion factor to convert quantitative values in copies/ml to IU/ml to allow for comparison of test results between laboratories that perform HIV-2 RNA viral load assays. Two fold serial dilutions of the reconstituted WHO HIV-2 IS ranging from 1,000 to 8 IU/ml were tested in replicates of three or four (Table 2). The average of measurements between 125 to 1,000 IU/ml provided a conversion factor of 13.7 copies/IU, which can be used to convert values for comparison of test results to that of other assays. Values below 100 IU/ml were beyond the linear range of the assay and were not reliable for use in the assignment of the conversion factor. With four (4) of four (4) replicates detected at 63 and 31 IU/ml and two (2) of four (4) replicates detected at 16 IU/ml, the LOD of the WRAIR qRT-PCR assay is estimated at 31 IU/ml for the WHO HIV-2 International Standard.

**Table 2:** International unit conversion based on replicate testing of serial dilutions of WHO HIV-2 International Standard.

IU/ml	Measured Copies/ml				Ave	SD	%CV	Copies/IU Conversion
**1000**	12,513	20,473	9,316	-	14,101	5,745	41%	14.1
**500**	7,325	3,450	10,975	6,075	6,956	3,128	45%	13.9
**250**	6,438	2,025	3,713	2,800	3,744	1,924	51%	15.0
**125**	2,163	1,363	1,900	413	1,460	773	53%	11.7
**63**	2,000	825	1,313	188	1,082	766	71%	
**31**	725	413	988	388	629	285	45%	
**16**	188	BLD	BLD	300	244	79	32%	
**8**	325	BLD	BLD	113	219	150	68%	
							**Ave**	**13.7**

BLD = below limit of detection.

### Limit of detection (LOD)

**Fig 5:**
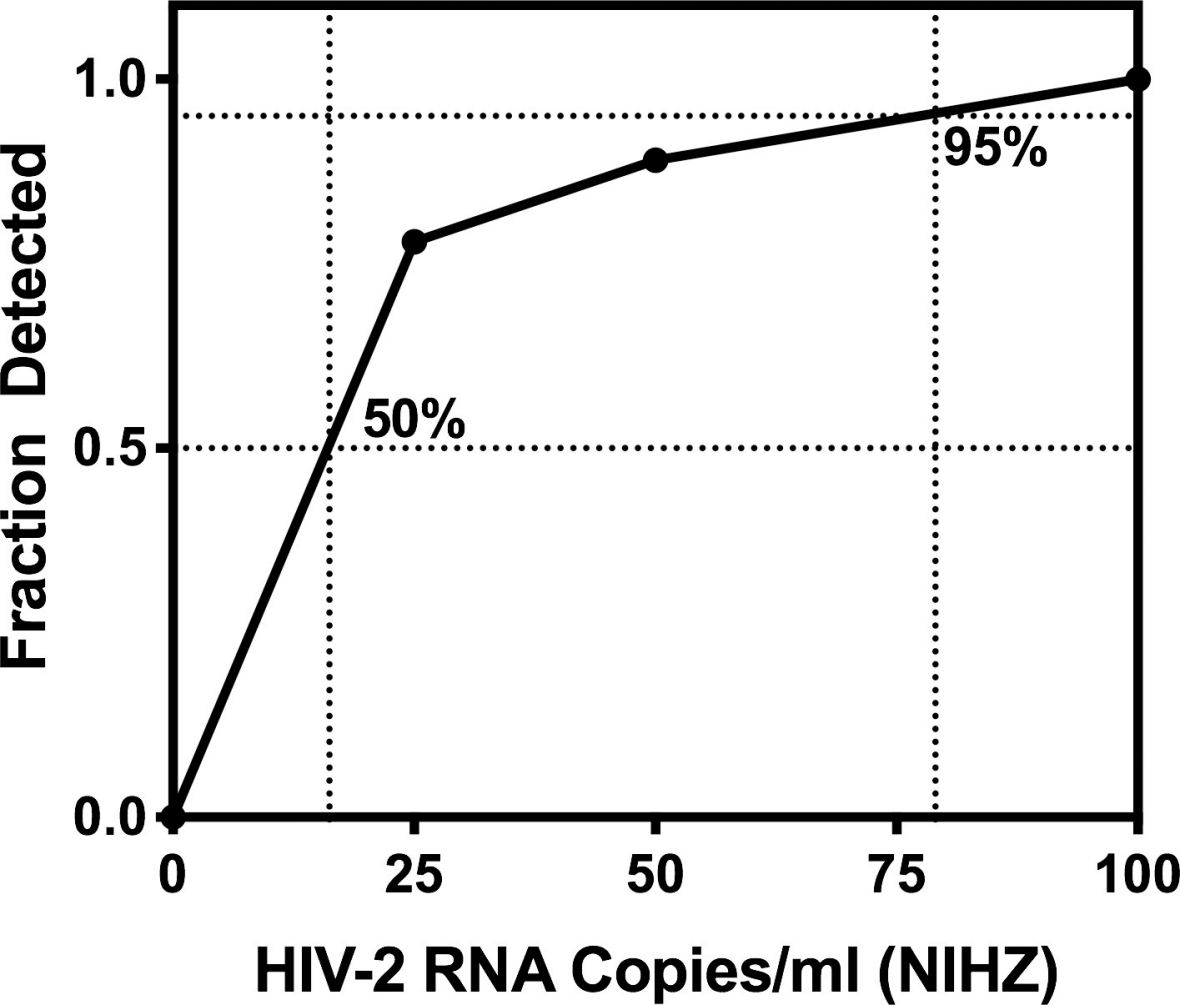
Limit of detection (LOD). Replicate testing of serial dilutions of HIV-2 NIH-Z isolate in BaseMatrix performed over three (3) assay runs (9 replicates total) were used to calculate the LOD at 95% (79 copies/ml) and 50% (16 copies/ml) confidence limits.

A more accurate measurement of the limit of detection for the WRAIR qRT-PCR assay was evaluated by replicate measurements of two-fold dilutions of the HIV-2 NIH-Z isolate ranging from 500 to 25 copies/ml. All nine of nine replicates were detected at and above 100 copies/ml, while eight of nine replicates were detected at 50 copies/ml, and seven of nine replicates at 25 copies/ml (Fig 5). Analysis of results indicate that the assay LOD based upon the NIH-Z strain is 79 copies/ml at 95% Confidence Interval (CI) and 16 copies/ml at 50% CI.

### Reproducibility

The reproducibility of the WRAIR qRT-PCR assay was assessed by triplicate testing of HIV-2 clinical samples (Boca Biolistics) over two runs (Table 3). RNA was detected in all three replicates for nine of twelve samples with viral loads ranging from 2.47 to 5.65 log_10_ copies/ml and SD between 0.05–0.46. The highest %CV was observed for Sample 9 which had a HIV-2 viral RNA value under 1,000 copies/ml. Two of the samples were not detected in the assay, and a third sample was detected in only one of three replicates.

**Table 3:** Assay reproducibility in clinical specimens.

ID	Test 1	Test 2	Test 3	Ave	SD
1	5.39	5.89	5.67	5.65	0.25
2	5.34	5.20	5.05	5.19	0.14
3	5.27	5.17	4.91	5.12	0.19
4	4.61	4.55	4.74	4.63	0.10
5	4.56	4.46	4.48	4.50	0.05
6	4.40	4.52	4.35	4.42	0.09
7	3.83	4.09	4.18	4.04	0.18
8	3.63	3.39	3.27	3.43	0.19
9	2.05	2.40	2.96	2.47	0.46
10	TND	TND	2.17	-	-
11	TND	TND	TND	-	-
12	TND	TND	TND	-	-

SD = Standard Deviation

TND = Target Not Detected

### Comparison to other qRT-PCR assays

The performance of the WRAIR qRT-PCR assay was compared to the SeraCare and HIV-2 Genesig (Primer Design) assays using the SeraCare PRF203 HIV-2 RNA Performance Panel (Table 4). The WRAIR result for the three highest titer panel members (>2.0 log_10_ copies/ml) were within 0.18 to 0.71 log_10_ of the SeraCare results and comparable to those of the Genesig assay. Panel member seven which was at 2.2 log_10_ copies/ml in the SeraCare assay and 1.65 log_10_ by the Genesig assay was not detected by the WRAIR assay. Of the seven panel members below 2.2 log_10_ copies/ml, three were Below Level of Quantitation (BLQ) and four were TND on the SeraCare assay while the Genesig assay detected three and were TND on four. The WRAIR assay was BLQ on 2 samples and TND on the other five.

**Table 4:** Comparison of three HIV-2 RNA viral load assays (log_10_ copies/ml) on the SeraCare HIV-2 PRF203 Performance Panel.

	SeraCare	Genesig	WRAIR
1	4.04	3.58	3.33
2	3.45	3.38	3.17
3	2.65	1.98	2.19
4	2.20	1.65	TND
5	BLQ	1.60	BLQ
6	TND	1.70	BLQ
7	BLQ	1.18	TND
8	BLQ	TND	TND
9	TND	TND	TND
10	TND	TND	TND
11	TND	TND	TND

BLQ = Below Limit of Quantification. TND = Target Not Detected.

Comparison of assay performance was also evaluated for the eight early infection samples from the SeraCare Cote d’Ivoire clinical sample set. All eight of the discrepant early infection samples which were HIV Ag/Ab combo reactive, but HIV-2 confirmatory/differentiation assay negative had HIV-2 RNA that was quantified by quantitative HIV-2 RNA reference assays and by the WRAIR qRT-PCR assay. One of these samples was below 100 copies/ml in both assays, while the remaining samples were between 100 and 587,000 copies/ml, with parallel results between the WRAIR and Reference Laboratory assays.

### Clinical samples

**Fig 6:**
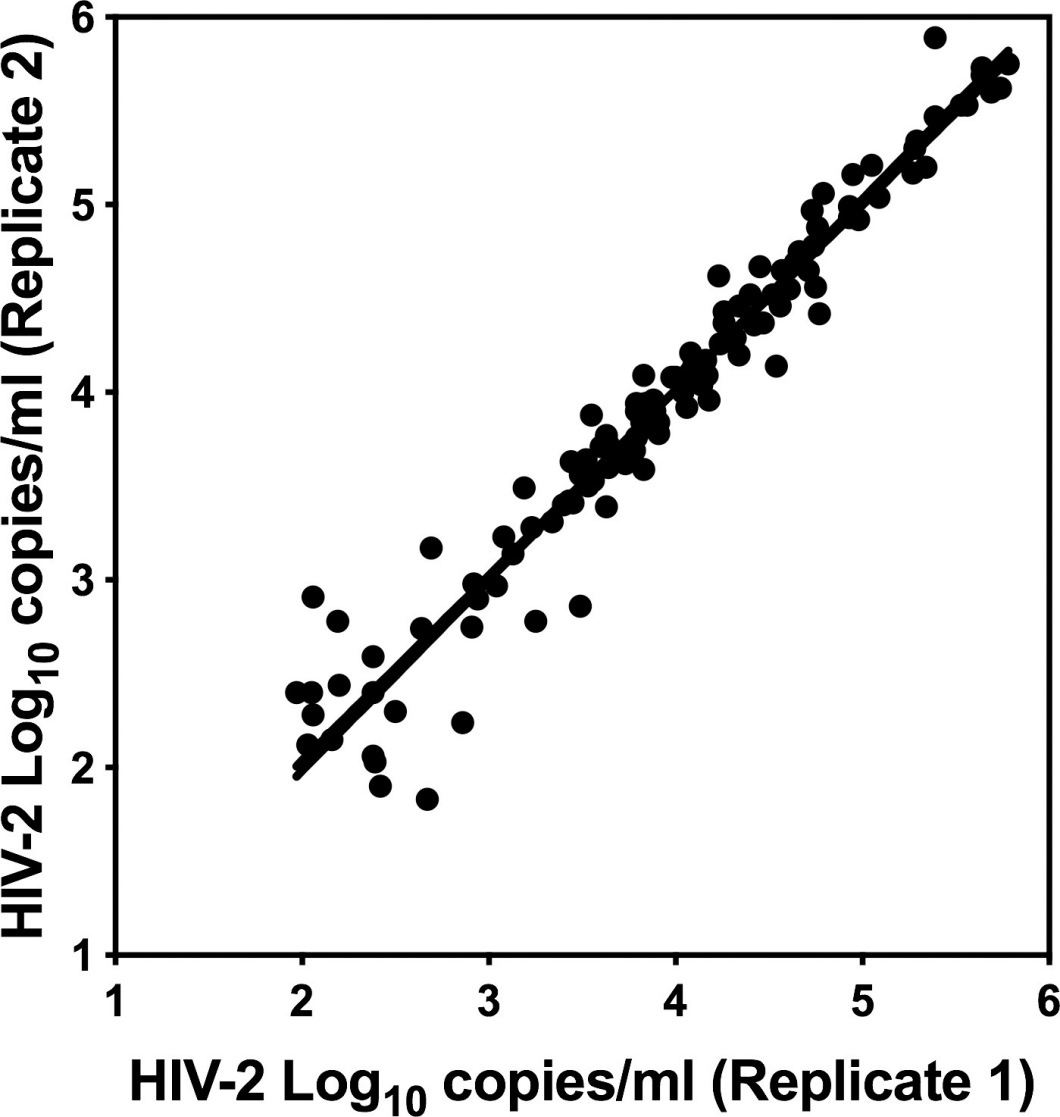
HIV-2 seropositive clinical specimens. Duplicate measurements of each sample showed consistent results, with the greatest variability observed for samples at or below 1,000 HIV-2 RNA copies/ml. Replicate values showed good correlation (Pearson r = 0.9723, p<0.0001) and linearity (*R*^2^ = 0.0.9454: Y = 0.9869*X + 0.0634). Of the 220 HIV-2 positive clinical specimens tested, 20 specimens (9.1%) were RNA positive in one of two replicates and 82 specimens (37.3%) did not have detected RNA in either replicate by the WRAIR assay.

Two hundred and twenty confirmed HIV-2 plasma samples from Cote d’Ivoire were tested in duplicate in the WRAIR qRT-PCR assay. Measured HIV-2 viral RNA values for 118 samples (53.6%) having replicate results ranged from 1.83 to 5.89 log_10_ HIV-2 RNA copies/ml. Replicate HIV-2 values from the clinical specimens showed significant correlation (Pearson r = 0.9723, p< 0.0001) and linearity (*R*^2^ = 0.9454) between each other showing good assay reproducibility (Fig 6) above 1,000 copies/ml. The mean of ratios between the first and second results for clinical samples (N = 22: 10%) at or below 1,000 copies/ml was 1.0075 with a range from 0.8208 to 1.4590; whereas, samples with viral loads greater than 1,000 had a mean ratio of 0.9997 ranging from 0.9140 to 1.2202. Thus, higher variability was observed at viral RNA levels below 1,000 copies/ml. This variability is also reflected in the twenty samples (9.1%) having measurable HIV-2 RNA in one of two replicates as the majority of these samples had HIV-2 viral RNA values under 350 copies/ml. Only 7.3% of samples demonstrated HIV-2 viral loads above 100,000 copies/ml and 36.8% had HIV-1 viral RNA values between 1,000 and 100,000 copies.ml. HIV-2 RNA was not detected by the WRAIR qRT-PCR assay in 82 (37.3%) samples.

## Discussion

A highly sensitive, specific and robust HIV-2 quantitative PCR assay, targeting the HIV-2 LTR, was developed for clinical monitoring of HIV-2 plasma viral load in infected individuals. Evaluation of the assay performance demonstrated a high degree of precision, specificity, sensitivity, and linearity across a wide dynamic range thus meeting performance guidelines for monitoring plasma viral load of HIV-2 infected individuals [40, 41].

The MS2 Internal Control, which was spiked into the extraction buffer, showed consistent recovery of RNA and reproducible amplification for each sample tested and allowed for monitoring of sample recovery and inhibition. A C_*T*_ value of more than 3 SD above the mean for the Internal Control indicated poor sample recovery or amplification and the sample was rejected as invalid. Preliminary attempts at multiplex amplification of MS2 and HIV-2 LTR resulted in interference and reduced sensitivity at low concentrations of HIV-2, possibly due to competition between primers and probes. More consistent results at low HIV-2 RNA concentrations were obtained when the amplifications were performed in separate reactions under identical temperature and cycling parameters. These conditions were incorporated into the final assay.

The primers, probes and amplification conditions provided efficient detection of all Group A and Group B isolates of HIV-2 tested. The Step-up amplification method employing five (5) cycles at a lower stringent annealing temperature followed by 40 cycles at higher stringency allowed for sensitive detection despite sequence diversity and should amplify all major HIV-2 groups in circulation. The specificity of the assay was demonstrated by absence of detectable HIV-2 RNA in negative controls (N = 127), plasma (N = 21) or EDTA blood (N = 50) from uninfected individuals; no cross reactivity with HIV-1, hepatitis B or hepatitis C viruses was observed.

The performance of the linearity panel, as well as that of the assay calibrators included with each assay, showed a high degree of linearity (*R*^2^ > 0.997) and reproducible cycle threshold values (2.2–7.6% CV), allowing for reliable measurement of HIV-2 plasma viral load across the dynamic range found in clinical samples. A manual assay platform was used for extraction and amplification resulting in acceptable but slightly lower precision and reproducibility than that achieved with an automated system. Consistent quantitative results were obtained within 0.3 logs for samples above 1,000 copies/ml. Although higher variance is observed for HIV-2 viral loads under 1,000 HIV-2 copies/ml, the precision of our assay is similar to that reported for other assays performed at the University of Washington and the New York State Department of Health Labs which are performed on automated platforms for sample processing and/or amplification [24, 25].

The HIV-2 Performance Panel PRF203 showed that the WRAIR qRT-PCR resulted in comparable HIV-2 quantitative values to those obtained with the SeraCare and Genesig Research Use Only assays. The LOD of the WRAIR assay based upon the NIH-Z viral stock (79 copies/ml) was comparable to the reported 50 copies/ml noted for the SeraCare assay which uses TaqMan chemistry targeting the HIV-2 *gag* sequence (unpublished). Similar to the WRAIR qRT-PCR assay, SeraCare and Genesig assays use external calibrators that are not subjected to RNA extraction and as such can result in an under-estimation of the RNA concentration due to the loss of RNA during extraction. As the Genesig calibrator is DNA, we used the NIH-Z viral stock of HIV-2 as a calibrator for the Genesig HIV-2 qRT-PCR assay in this study. Implementing a calibrator that is extracted along with the test sample is expected to improve quantitative values obtained in our assay.

Standardization of the assay against the WHO HIV-2 IS allows comparison of the WRAIR qRT-PCR results to those of other laboratories. The conversion factor was tentatively established at 13.7 copies/IU based on the mean results of four different measurements that varied from 11.7 to 15.0 copies/IU. Unfortunately, the low concentration of the WHO HIV-2 IS (1,000 IU/ml) and the annual limit on quantity available made it difficult to perform sufficient replicate testing to allow for more accurate assignment of a conversion factor. The results for serial dilutions of the WHO HIV-2 International Standard (Table 2) demonstrated that all replicates at 31 IU/ml were detected while 50% of the replicates at 16 and 8 IU/ml were detected. Assignment of a LOD based upon International Units was not possible due to insufficient number of replicates, but did permit an estimation of LOD at 31 IU/ml. Our estimated LOD is comparable to that of 53 IU/ml for the HIV-2 assay reported for the University of Washington, or the 31 IU/ml (0.2 ml sample) at the New York State Public Health Laboratories [25, 26]. Of note when the estimated LOD at 31 IU/ml is converted to copies per ml using our conversion factor of 13.7, the LOD in copies per ml is 425. This LOD differs from the LOD obtained using the NIH-Z HIV-2 viral stock. LOD determinations will differ depending upon the reference source with an increase in disparity when using materials quantitated by differing methods. The NIH-Z viral stock concentration is based upon electron microscopy count whereas the WHO International Standard concentration is determined by averaging values from multiple assays. Our assay is capable of detecting ten HIV-2 input copies based upon our RNA calibrator. Factoring in sample volume, this value equates to a LOD of 125 copies/ml. In our assay specimens having HIV-2 viral RNA concentrations under 1,000 copies/ml have higher variability and as the viral RNA approach 100 copies/ml, our assay is more likely to have a not detected result.

The performance of our assay could be improved by increasing the amount of sample tested, use of an automated extraction instrument and the inclusion of a second amplification target. The LOD of our assay is limited by sample volume (0.4 ml) and the volume of RNA (10 μl/ 50 μl) included in the amplification reactions. Further improvements in assay sensitivity could include alternative extractions methods allowing for extraction from a larger volume of plasma and/or real-time PCR instrumentation having larger volume capacity wells that allow for great input volume. The addition of a second target region in the *gag* gene may also improve the performance of a future version of our assay and align it with recommendations that HIV-1 quantitative assays include two target regions. Inclusion of two amplification target regions in the assay will also improve the detection of HIV-2 viral RNA in specimens having a nucleotide base mismatch and may allow for improved detection and quantification of HIV-2 Group B classified viruses as one of two group B isolates tested in this study was under-quantified. The performance of a dual-target assay for HIV-2 viral load is reported by Bertine et.al. (29). HIV-2 Group B isolates are very rare in the United States and we were only able to include two B isolates in our study. Additional Group B isolates would be required to validate the performance of future assays.

The clinical performance of the WRAIR qRT-PCR was evaluated on 220 clinical plasma samples from confirmed HIV-2 infected individuals from Cote d’Ivoire. Although the samples provided were noted as serologically reactive for HIV-2 by HIV type differentiation assays, 42% of the samples when tested in duplicate did not demonstrate detectable HIV-2 RNA by our assay. Nevertheless our results are comparable with those reported for other laboratory developed HIV-2 RNA quantitative assays which showed that plasma HIV-2 RNA levels in viremic individuals tend to be low (2.8 log_10_ copies/ml on average) with only 6% of viremic individuals over 1,000 copies/ml, and below level of detection in 28.0 to 46.5% of confirmed HIV-2 infections [28, 29, 31, 32]. The high proportion of undetectable viral load among the clinical samples tested is similar to that reported in other studies in Europe and Africa. Low viremia has been reported to result in lower transmission and pathogenicity of the virus [42]. Diagnosis later in infection may result in 70% of the HIV-2 infected individuals having undetectable viral RNA.

Technical issues that could contribute to the lower viral RNA measurements may also include unreported use of antiretroviral drugs as no information was provided with our clinical samples as to length of time from infection (1) or additional loss of RNA signal related to the transport and long term storage of the samples used in our study.

The high levels of undetectable viral load in HIV-2 infected individuals limits the usefulness of quantitative plasma HIV-2 RNA PCR assays for diagnosis or confirmation of infection. Alternative approaches, such as testing of whole blood or PBMCs for HIV-2 total nucleic acid (TNA: RNA and DNA) may be more appropriate for those applications [26, 28, 41]. Our studies with HIV-1 infections demonstrated that the HIV-1 TNA is readily detected in ART treated HIV-1 infected individuals even in the absence of detectable plasma viral load [43]. Indeed, several studies of HIV-2 infected individuals have shown that viral nucleic acid can be readily detected by TNA testing of whole blood and PBMCs even when plasma viral load is very low or undetectable [25, 26, 28].

## Conclusion

The results of our studies are consistent with those of previous studies with plasma samples from serologically confirmed HIV-2 infected individuals which found low plasma viral load levels, with 40–70 percent of individuals below level of detection. The WRAIR HIV-2 qRT-PCR assay has a comparable LOD based upon IU/ml to the HIV-2 qRT-PCR assays performed at University of Washington and NY State Health Department. The results of our studies show that the WRAIR qRT-PCR assay provides a convenient, sensitive, specific, and reproducible measure of HIV-2 viral RNA in plasma and is suitable for use in guiding treatment, monitoring of therapy and following disease progression in HIV-2 infected individuals.
